# Tree Species Diversity and Forest Edge Density Jointly Shape the Gut Microbiota Composition in Juvenile Great Tits (*Parus major*)

**DOI:** 10.3389/fmicb.2022.790189

**Published:** 2022-03-09

**Authors:** Evy Goossens, Roschong Boonyarittichaikij, Daan Dekeukeleire, Lionel Hertzog, Sarah Van Praet, Frank Pasmans, Dries Bonte, Kris Verheyen, Luc Lens, An Martel, Elin Verbrugghe

**Affiliations:** ^1^Department of Pathology, Bacteriology and Avian Diseases, Faculty of Veterinary Medicine, Ghent University, Merelbeke, Belgium; ^2^Department of Clinical Sciences and Public Health, Faculty of Veterinary Science, Mahidol University, Nakhon Pathom, Thailand; ^3^Terrestrial Ecology Unit, Department of Biology, Ghent University, Ghent, Belgium; ^4^Forest & Nature Lab, Department of Environment, Ghent University, Ghent, Belgium

**Keywords:** great tits (*Parus major*), faeces, microbiota, tree species diversity, forest fragmentation

## Abstract

Despite the microbiome’s key role in health and fitness, little is known about the environmental factors shaping the gut microbiome of wild birds. With habitat fragmentation being recognised as a major threat to biological diversity, we here determined how forest structure influences the bacterial species richness and diversity of wild great tit nestlings (*Parus major*). Using an Illumina metabarcoding approach which amplifies the 16S bacterial ribosomal RNA gene, we measured gut microbiota diversity and composition from 49 great tit nestlings, originating from 23 different nests that were located in 22 different study plots across a gradient of forest fragmentation and tree species diversity. Per nest, an average microbiome was determined on which the influence of tree species (composition and richness) and forest fragmentation (fragment area and edge density) was examined and whether this was linked to host characteristics (body condition and fledging success). We found an interaction effect of edge density with tree species richness or composition on both the microbial richness (alpha diversity: Chao1 and Shannon) and community structure (beta diversity: weighted and unweighted UniFrac). No significant short-term impact was observed of the overall faecal microbiome on host characteristics, but rather an adverse effect of specific bacterial genera on fledging success. These results highlight the influence of environmental factors on the microbial richness as well as the phylogenetic diversity during a life stage where the birds’ microbiota is shaped, which could lead to long-term consequences for host fitness.

## Introduction

During the past two decades, the gut microbiome has attracted considerable research attention because of its role in a host’s physiology and health status. The microbiota of the gastrointestinal tract plays a fundamental role in food digestion, pathogen defence, stimulation of the immune system, gut and central nervous system functioning, life-history traits and behaviour (Macpherson and Harris, 2004; O’Hara and Shanahan, 2006; Kamada et al., 2013; Cenit et al., 2017; Macke et al., 2017; Grizotte-Lake et al., 2018). Despite its pivotal role in host health, research on the gut microbiota of avian species has lagged behind mammalian research and is dominated by studies of agriculturally important birds as well as birds of conservation interest (Grond et al., 2018). In wild bird populations, studies examining the gastrointestinal flora are rather scarce and mostly focusing on juvenile birds of which the gut microbiota is still undergoing substantial changes (Zhu et al., 2017; Teyssier et al., 2018a).

Initially the gut microbiota is shaped by external bacteria present in the nesting environment and by parental feeding (Hird et al., 2014; Ding et al., 2017; Teyssier et al., 2018a). Food-associated microbial communities may, however, vary by location and the food quality can pose a differential selection pressure on the gut microbiota (Grond et al., 2018; Davidson et al., 2020). As such, variation in habitat can determine microbiota composition, which may have implications on nestling body condition and long-term consequences for host fitness. For example, Teyssier et al. (2018b) showed that adult house sparrows from urban areas hosted microbial communities with lower diversity and fewer metabolic functions compared to rural populations.

In temperate zone forests, tree species composition and diversity shape arthropod abundance, and thus the food availability for insectivorous forest birds (Naef-Daenzer, 2000; Fuentes-Montemayor et al., 2012; Shutt et al., 2018). In addition, other environmental factors including forest fragmentation also impact the availability of food resources (Burke and Nol, 1998; Zanette et al., 2000). Great tits (*Parus major*) were shown to feed their nestlings less caterpillars in small fragments compared to larger fragments (Bueno-Enciso et al., 2016). The effects of forest fragmentation can, however, depend on the local tree species composition. In northern Belgium, breeding success of great tits decreases with fragment area in *Fagus sylvatica* forests but does not vary with fragment size in insect-rich *Quercus robur* forests (Dekeukeleire et al., 2019). It can thus be expected that habitat characteristics such as tree species composition and forest fragmentation also jointly affect the avian gut microbiota.

In this study, we assessed the individual and interacting effects of environmental factors such as tree species (composition and richness) and forest fragmentation (fragment area and edge density) on the gut microbiota of wild birds. We use great tits, an insectivorous forest bird during breeding season, as a model species by sampling the gut microbiota in 49 great tit nestlings originating from 23 different nests to determine the average nest microbiome. The nests were located in 22 different forest fragments in the south of Ghent, northern Belgium, with a varying fragmentation gradient and tree species composition. Taking into account both forest composition and forest fragmentation allowed us to address the question whether alpha and beta diversity are shaped by forest structure and whether it has consequences for bird health and fitness. Previously, it has been shown that body condition of great tits is an important factor for bird fitness, especially for bird survival after fledgling (Monrós et al., 2003; Rodríguez et al., 2016; Teyssier et al., 2018a). With the transition from nestling to fledgling being a key moment in the development of altricial birds and with gut microbiota characteristics and nestling body condition in great tits being linked to each other (Teyssier et al., 2018a), we also explored the hypothesis that forest-driven changes in the microbiome can affect important host parameters including body condition and bird survival after fledgling. More specifically, we tested (1) if tits located in areas with greater tree species richness and diversity displayed higher faecal microbial richness and microbial community structure, (2) if habitats with larger areas defined as forest edges, which are associated with a greater abundance and diversity of arthropods, were associated with higher microbial diversity and (3) whether this affects host characteristics including body condition and fledging success of the great tits.

## Materials and Methods

### Study Site

**FIGURE 1:**
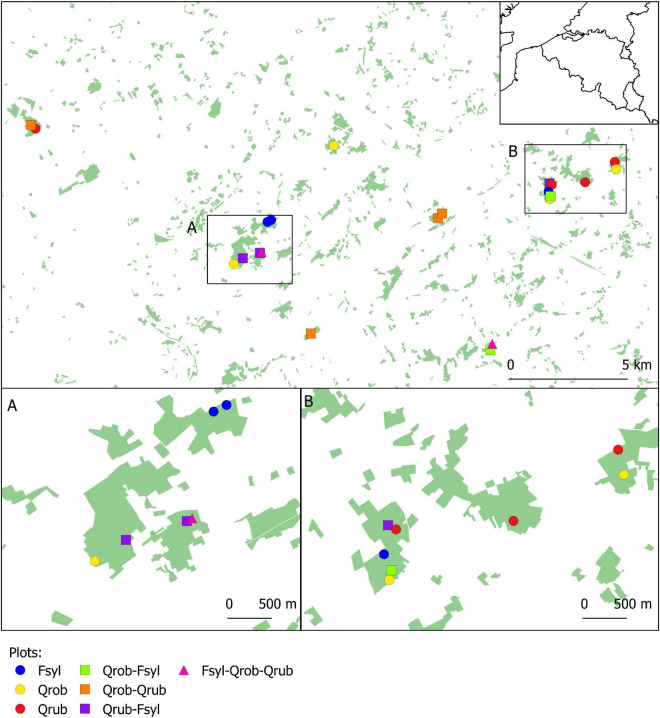
Map showing the location of all study plots. The 22 study plots are established in forest fragments with varying size and tree layer. Every study plot is represented by a coloured symbol corresponding to its respective tree species composition. Fsyl, European Beech (*Fagus sylvatica*, L); Qrob, Pedunculate Oak (*Quercus robur*, L); Qrub, Red Oak (*Quercus rubra*, L).

We performed a gut microbiota study of 23 great tit nests in 22 study plots (30 × 30 m) located in 11 mature (>60 years) deciduous forest fragments in the south of Ghent (coordinates: 50°57′19″N, 3°43′31″E), northern Belgium (Figure 1; see De Groote et al., 2017 for more details). These study plots were established in 2014 to study effects of tree species diversity and forest fragmentation on food web dynamics (Hertzog et al., 2019). Plots varied in tree species composition [Pedunculate oak (*Q. robur*), Red oak (*Quercus rubra*), and Beech (*F. sylvatica*) in monocultures, two species mixtures or three species mixtures] (Figure 1 and Supplementary Table 1). Plots of all tree species compositions are replicated along a fragmentation gradient, with some plots being situated in larger fragments far from the forest edge, and other in smaller forest fragments, close to the forest edge. All plots are located in forest stands with similar land-use history (continually wooded since at least 1850), management history (mature stands where no forestry management took place in the last decade) and soil (dry sandy loam).

As explained in Dekeukeleire et al. (2019), 12 fragmentation measures were collected (Supplementary Table 2) of which fragment-level forest area and plot-level edge density were selected as key components of habitat fragmentation (Ewers et al., 2007; Fisher and Lindenmayer, 2007). Fragment area was calculated as the total surface area of the forest fragment in which a study plot is situated (range: 10.74–90.36 ha), based on detailed land use GIS layers (Vriens et al., 2011; Dekeukeleire et al., 2019). Edge density quantifies the intensity of edge effects on individual plots, with higher values being characteristic for more fragmented forests. Edge density was calculated as the total length of all edges of the forest with other land use classes (e.g., agricultural land or residential areas) within a radius of 300 m of the plot (range: 655–2932.40 m) (Dekeukeleire et al., 2019). Previously these plots were used to analyse the effects of forest fragmentation and tree species composition on the breeding performance and body condition of Great and Blue tits (Dekeukeleire et al., 2019).

### Sample Collection

For full details on the field work procedures we refer to Dekeukeleire et al. (2019). Briefly, at the corner of each plot, four standard nest boxes for great tits (dimensions 23 × 9 × 12 cm, entrance 32 mm) were installed at a height of 1.5 m, in the autumn of 2014. During the breeding season (April–June 2015), all nest boxes were checked at least twice a week to determine occupancy by great tits and collect breeding data. At 14–15 days of age, the juveniles were fitted with a metal ring from the Belgian Ringing scheme, measured [weight (g) and tarsus length (mm)] and a faecal sample was collected. The body condition was calculated using the scaled-mass index (SMI). This adjusts the mass of all individuals to that which they would have obtained if they all had the same body size, using the equation of the linear regression of ln-body mass on ln-tarsus length estimated by type-II (standardised major axis; SMA) regression (Peig and Green, 2009; Supplementary Table 3). Faecal samples from 49 great tit nestlings, originating from 23 different nests were collected by placing the individual animals in a sterilised cotton bag. The birds were regularly checked to see if they defecated, with a maximum of a 30 min incubation period. If the birds defecated, the faecal samples were collected into Eppendorf tubes while wearing sterile gloves. All samples were kept in sterile Eppendorf tubes at -20°C until further analysis. The individual samples (*n* = 49) were sequenced and analysed on nest level (*n* = 23).

### DNA Extraction

Total community DNA was isolated from great tit faecal samples (*n* = 49) using the CTAB method modified from Kowalchuk et al. (1999) and Griffiths et al. (2000). Briefly, we added 0.5 ml CTAB buffer (hexadecyltrimethylammonium bromide 5% (w/v), 0.35 M NaCl, 120 nM K_2_HPO_4_) and 0.5 ml phenol-chloroform-isoamyl alcohol (25:24:1). The mixture was homogenised by grinding (2×) with 0.5 g unwashed glass beads (Sigma-Aldrich, Overijse, Belgium) in a bead beater (1.5 min, 22.5 Hz; TissueLyser; Qiagen, Hilden, Germany) with a 30 s interval between shakings. After centrifugation (10 min, 8000 *g*), 300 μl of the supernatant was transferred to a new tube. A re-extraction from the remaining content was performed by adding 0.25 mL CTAB buffer. After homogenisation as described above, samples were centrifuged (10 min, 8000 *g*) and 300 μl of supernatant was added to the first 300 μl. An equal volume (600 μL) of chloroform-isoamyl alcohol (24:1) was added to the supernatant collected in order to remove the phenol from the samples. The mixture was further centrifuged at 16 000 *g* for 10 s and the aqueous phase was transferred to a new Eppendorf tube. Nucleic acids were precipitated with two volumes of PEG-6000 solution [polyethyleenglycol 30% (w/v), 1.6 M NaCl] for 2 h at room temperature. After centrifugation (20 min, 13000 *g*), the pellet was rinsed with 1 ml of ice-cold, 70% (v/v) ethanol. The pellet was dried and resuspended in 100 μl RNAse free water. The quality and the concentration of the DNA was examined spectrophotometrically (NanoDrop, Thermo Scientific, Waltham, MA, United States). The isolated DNA from the individual samples (*n* = 49) were individually sequenced, but analysed on nest level (*n* = 23).

### PCR Amplification and High-Throughput Sequencing

Following the recommendations of Klindworth et al. (2013), the V3-V4 hypervariable region of the 16s rRNA gene was amplified using the gene-specific primers S-D-Bact-0341-b-S-17 (5′-CCTACGGGNGGCWGCAG-3′) and S-D-Bact-0785-a-A-21 (5′-GACTACHVGGGTATCTAATCC-3′). A 25 μL PCR reaction contained 2.5 μL DNA (∼20 ng/μL), 0.2 μM of forward and reverse primers and 12.5 μL 2× KAPA HiFi HotStart ReadyMix (Roche, Diegem, Belgium). PCR conditions were as follows: initial denaturation at 95°C for 3 min, followed by 25 cycles of 95°C for 30 s, 55°C for 30 s, 72°C for 30 s, and a final extension at 72°C for 5 min. Subsequently, the PCR products were purified using CleanNGS beads (CleanNA) and the DNA quantity and quality was analysed spectrophotometrically (NanoDrop) and by agarose gel electrophoresis (1.5% agarose). In a second PCR, dual indices were attached to the 16S V3-V4 fragment. This 50 μL PCR reaction contained 5 μL of purified PCR product, 2× KAPA HiFi HotStart ReadyMix (25 μL) and 2.5 μl index primer 1 (N7xx) (10 μM stock) and index primer 2 (S5xx) (10 μM stock). PCR conditions were as follows: initial denaturation at 95°C for 3 min, followed by eight cycles of 95°C for 30 s, 55°C for 30 s, 72°C for 30 s and a final extension at 72°C for 5 min. The final PCR products were purified and the concentration was determined using the Quantus fluorimeter (Promega, Leiden, Netherlands). The final barcoded libraries were combined to an equimolar 5 nM pool and sequenced with 30% PhiX spike-in using the Illumina MiSeq v3 technology (2 × 300 bp, paired-end) at the Oklahoma Medical Research center (Oklahoma City, OK, United States).

### Bioinformatic Processing of 16S rRNA Data

Demultiplexing of the amplicon dataset and deletion of the barcodes was done by the sequencing provider. Quality of the raw sequence data was checked with the FastQC quality-control tool (Babraham Bioinformatics, Cambridge, United Kingdom^1^) followed by initial quality filtering using Trimmomatic v0.38 by cutting reads with an average quality per base below 15 using a 4-base sliding window and discarding reads with a minimum length of 200 bp (Bolger et al., 2014). The paired-end sequences were assembled and primers were removed using PANDAseq (Masella et al., 2012), with a quality threshold of 0.9 and length cut-off values for the merged sequences between 390 and 430 bp. Chimeric sequences were removed using UCHIME (Edgar et al., 2011). Open-reference operational taxonomic unit (OTU) picking was performed at 97% sequence similarity using USEARCH (v6.1) (Edgar, 2010) and OTU taxonomy was assigned against the Silva database (v128, clustered at 97% identity) (Quast et al., 2013). OTUs with a total abundance below 0.01% of the total sequences were discarded (Bokulich et al., 2013), resulting in an average of approximately 13288 reads per sample. Alpha rarefaction curves were generated using the QIIME “alpha_rarefaction.py” script and a subsampling depth of 7800 reads was selected, which was used for all subsequent analyses. Any sequences of mitochondrial or chloroplastic origins were removed before further analysis.

### Statistical Analyses

All statistical analyses were performed in R (v3.5.1) (R Core Team, 2018). Due to non-convergence of LMM when analysing the microbiome on bird level (*n* = 49), and to normalise for a possible nestbox effect and difference in the number of individuals sampled per nest (Supplementary Table 3), nestlings sampled from the same nest box were treated as one biological replicate by averaging the OTUs per nest. As such, the microbiome analysis was performed on nest level (*n* = 23). Microbiota alpha diversity (Chao1 richness estimator and Shannon diversity estimator) measures were calculated using the *phyloseq* package (McMurdie and Holmes, 2013) and showed a Gaussian distribution. Linear models were used to test the effect of different forest parameters (tree species richness, tree species composition, fragment area, or egde density) on the microbial alpha diversity, as well as the resulting effects on fledging success (number of fledglings/number of nestlings) or fledgling body condition (SMI). In accordance with the OTU’s, the fledgling SMI and fledging success were averaged on nest level (Supplementary Table 3). Models were run for each alpha diversity metric separately. In each model the explanatory variables were: a tree species measure (composition or richness), a measure of forest fragmentation (edge density or fragment area) and the interaction between both. As established in Dekeukeleire et al. (2019), there is a high degree of collinearity between the individual measures. Therefore, all models were run with one tree species measure (composition or richness; categorical variable) and one fragmentation measure (fragment area or edge density; continuous variable; Pearson’s *R* = −0.43) at a time. For all models, significant effects were determined by ANOVA (type I sum of squares). The significance of the fragmentation effects for the different tree richness or tree composition levels was assessed using the addSE packages (Hertzog, 2018). The effect of microbiota diversity on host characteristics was assessed by including either nestbox fledging success (binomial distribution) or average fledgling SMI per nestbox (Gaussian distribution) as response variable. In models including body condition, the number of nestlings was included as an additional fixed covariate.

Beta diversity was studied using *phyloseq*, taking Jaccard (presence/absence), Bray–Curtis (presence/absence, as well as abundance), unweighted UniFrac (phylogenetic distance) and weighted UniFrac (phylogenetic distance, as well as abundance) dissimilarities into account. The effect of either tree species composition or tree species richness and forest fragmentation (fragment area or edge density) on the microbial community composition was assessed *via* permutational multivariate analysis of variance (PERMANOVA) using the *adonis* function from the *vegan* package (Dixon, 2003). The effect of the microbial community composition on either nestbox fledging success or average fledgling SMI per nestbox, was assessed using linear models. Models were run for each beta diversity metric separately, with either fledging success or body condition as response variable, and the first two principal coordinates from the beta diversity dissimilarity matrix as explanatory variables.

DESeq2 analysis was performed on the genus level abundance data to identify bacterial genera that (1) are driving the difference in microbial community composition in monocultures with varying edge densities by taking into account the interaction between the tree species richness and the edge density and (2) are linked to host characteristics by considering either average fledgling SMI per nestbox or nestbox fledging success. Significant differences were obtained using a Wald test followed by a Benjamini–Hochberg multiple hypothesis correction. For all tests, a *p*-value < 0.05 was considered significant.

### Ethical Considerations

Bird captures and handling were carried out under licence and guidelines of the Belgian Ringing Scheme and the Flemish authorities (Agentschap voor Natuur en Bos; ANB/BL-FF/V15-00034). All trapping and sampling protocols of great tits were approved and permitted by the Ethical Committee VIB (the Flanders Institute for Biotechnology) Ghent site (EC2015-023) with permissions of all site owners.

## Results

### Taxonomic Composition of Microbiota

**FIGURE 2:**
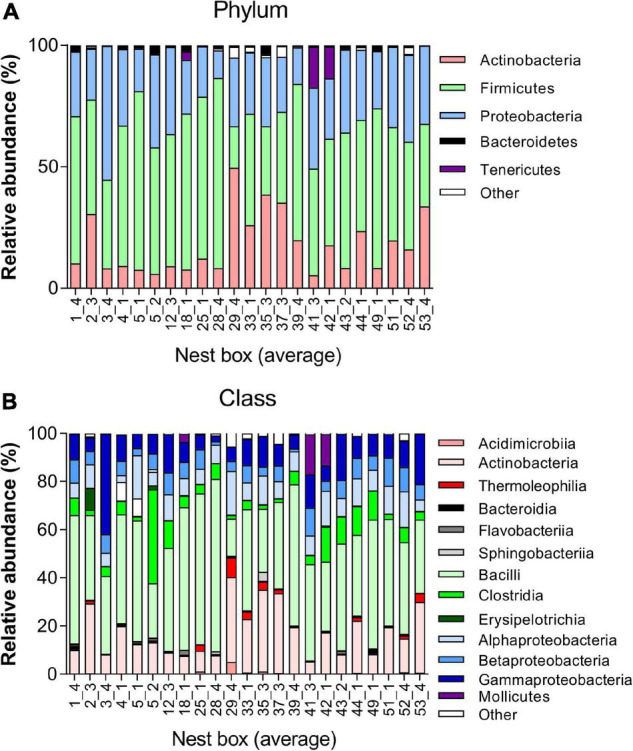
Taxonomy plot of gut microbiota of juvenile great tits. Composition analysis per nestbox at **(A)** Phylum level showing the five most abundant phyla and **(B)** Class level showing the 13 most abundant classes.

The microbial taxonomic composition of the juvenile great tit faecal samples was characterised by a predominance of the phyla Firmicutes (50.96 ± 14.63%), Proteobacteria (27.58 ± 8.44%), and Actinobacteria (17.90 ± 12.38%). Bacteroidetes (1.16 ± 1.09%), Tenericutes (1.49 ± 4.33%), and other phyla (0.91 ± 1.28%) showed a lower abundance (Figure 2 and Supplementary Datas 1, 2). When selecting the 5 most abundant genera in all nests, the genera *Staphylococcus*, *Bacillus*, *Lactobacillus*, and *Carnobacterium* belonging to the Class Bacilli, together with genus *Burkholderia-Paraburkholderia* belonging to the Class Betaproteobacteria were most prevalent in the great tit microbiome (Supplementary Data 3).

### Influence of Tree Species Diversity and Forest Fragmentation on the Faecal Microbial Composition of Great Tits

#### Alpha Diversity

**FIGURE 3:**
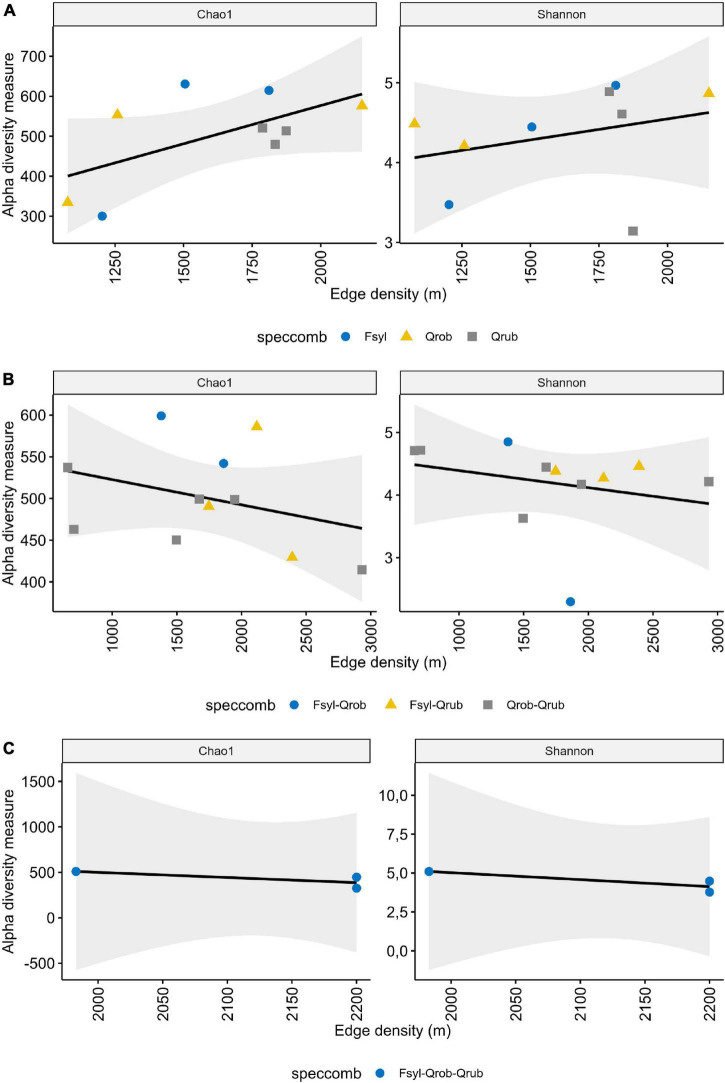
Influence of edge density and tree diversity on alpha diversity. Shown is the alpha diversity depending on the edge density in **(A)** monoculture, **(B)** 2 species, and **(C)** 3 species forests. Each point represents one nestbox, which is considered as one biological replicate. Fsyl, European Beech (*Fagus sylvatica*, L); Qrob, Pedunculate Oak (*Quercus robur*, L); Qrub, Red Oak (*Quercus rubra*, L). Chao1: estimated OTU richness and Shannon: estimated community diversity.

Faecal samples harboured on average 456 ± 92.89 OTUs (min = 268, max = 603 observed OTUs). When taking the interaction between tree species and edge density into account, a significant influence was observed on the alpha diversity, indicating an edge density effect on microbial richness with a direction depending on the particular tree species richness or composition (Supplementary Table 4). A significant interaction effect of tree species richness with edge density on Chao1 (ANOVA: *F*-value = 4.157; *DF* = 2; *p*-value = 0.034) and tree species composition with edge density on the Shannon diversity (ANOVA: *F*-value = 7.717; *DF* = 6; *p*-value = 0.004) was observed (Supplementary Table 4). More specifically a significant increase in OTU richness with edge density was observed in monoculture forests [Figure 3 and Supplementary Table 4 (AddSE): Chao1 slope: 0.191, 95% CI: 0.040/0.342]. Fragment area as a fragmentation metric in combination with tree species diversity did not significantly impact the alpha diversity (Supplementary Table 4).

#### Beta Diversity

Permutational multivariate analysis of variance analysis on the beta diversity showed that the interaction between tree species composition and edge density significantly affected the UniFrac dissimilarity indexes, and accounted for 36.2% (unweighted UniFrac, *p* = 0.041) to 37.4% (weighted UniFrac, *p* = 0.055) of the variation observed between the nests. The combined effects of tree species richness and edge density could explain 23.2% (unweighted UniFrac, *p*-value = 0.001) to 19.7% (weighted UniFrac, *p*-value = 0.007) of the variation (Supplementary Table 5 and Supplementary Figure 1). The Jaccard and Bray–Curtis indexes were not significantly influenced by tree species and/or fragmentation metrics (*p*-value > 0.05).

#### Abundance Changes of Bacterial Genera That Are Linked to the Interaction Effect of Edge Density With Tree Species Richness

Using DESeq2 analysis, 12 genera were identified as having a significant (adjusted *p*-value < 0.05) differential abundance in monocultures when the edge density varies (Table 1). When selecting the most abundant genera (baseMean > 100) *Lactococcus* (Class: Bacilli) was show to be most prevalent in monoculture plots characterised by a small edge density. *Pseudarthrobacter*, *Arthrobacter* (Class: Actinobacteria), and *Erysipelatoclostridium* (Class: Erysipelotrichia) showed a higher abundance in monoculture plots characterised by a high density of edges (Table 1).

**TABLE 1:** Differentially abundant genera linked to an edge effect in monoculture plots.

Phylum	Class	Order	Family	Genus	baseMean	log_2_ fold change	lfcSE	*p*adj
Actinobacteria	Actinobacteria	Micrococcales	Microbacteriaceae	*Ambiguous_taxa*	95.76019	0.00757	0.00194	0.00500
Actinobacteria	Actinobacteria	Micrococcales	Microbacteriaceae	*Frigoribacterium*	32.34403	0.00633	0.00176	0.00841
Actinobacteria	Actinobacteria	Micrococcales	Micrococcaceae	*Micrococcus*	79.87509	−0.0045	0.00107	0.00195
Actinobacteria	Actinobacteria	Micrococcales	Micrococcaceae	*Pseudarthrobacter*	328.35829	0.0102	0.00186	<0.00001
Actinobacteria	Actinobacteria	Micrococcales	Micrococcaceae	*Arthrobacter*	262.31776	0.00926	0.00181	0.00003
Actinobacteria	Actinobacteria	Micrococcales	Cellulomonadaceae	*Oerskovia*	14.90286	0.00845	0.00257	0.02389
Actinobacteria	Actinobacteria	Corynebacteriales	Dietziaceae	*Dietzia*	33.86723	0.01118	0.00304	0.00728
Proteobacteria	Betaproteobacteria	Burkholderiales	Burkholderiaceae	*Ralstonia*	59.05551	0.00392	0.00105	0.00680
Firmicutes	Bacilli	Lactobacillales	Streptococcaceae	*Lactococcus*	155.09241	−0.00488	0.00156	0.03341
Firmicutes	Erysipelotrichia	Erysipelotrichales	Erysipelotrichaceae	*Erysipelatoclostridium*	123.11971	0.00567	0.00180	0.03341
Firmicutes	Clostridia	Clostridiales	Lachnospiraceae	*[Eubacterium] hallii group*	7.19277	−0.00521	0.00168	0.03499
Actinobacteria	Actinobacteria	Micrococcales	Microbacteriaceae	*Microbacterium*	42.4276	0.00376	0.00100	0.00680

*The faecal microbiome of great tits nesting in monoculture forest fragments were analysed using DESeq2 analysis to identify differentially abundant taxa along a gradient of edge density. Significant differences in genus level abundance (adjusted p-value < 0.05) in the faecal microbiota from great tits along a gradient of edge density. The taxonomic classification, the baseMean, the log_2_ fold change, the log fold change Standard Error (lfcSE), and adjusted p-values of the DESeq2 normalised abundance of each genus are shown. The log2 fold change indicates the difference in bacterial abundance in monoculture plots if the edge density changes with one measure.*

### Influence of the Microbiome on Body Condition and Fledging Success of Great Tits

#### Microbial Diversity

The great tits showed an overall SMI ± SD of 18.06 ± 1.61 and an overall fledging success ± SD of 80.72 ± 29.38%. On nest level, the global microbiome diversity did not affect the host characteristics as neither the alpha diversity, nor the beta diversity significantly impacted the average fledgling SMI per nestbox or nestbox fledging success (Supplementary Tables 6, 7).

#### Abundance Changes of Bacterial Genera That Are Linked to the Body Condition or Fledging Success

On nest level, no specific genera were associated with the average SMI per nestbox. When examining the driving genera that are linked to fledging success, 19 genera were negatively associated with fledging success (Table 2). Nestboxes having a higher fledging success were associated with decreased numbers (baseMean > 100) of *Brachybacterium*, *Brevibacterium* (Class: Actinobacteria), *Serratia* (Class: Gammaproteobacteria), *Sphingomonas* (Class: Alphaproteobacteria), *Carnobacterium, Jeotgalicoccus* (Class*:* Bacilli), and *Tyzzerella 3* (Class: Clostridia), with fold changes (log_2_) that vary between −2.12 and −30.00 (Table 2).

**TABLE 2:** Differentially abundant genera linked to fledging success.

Phylum	Class	Order	Family	Genus	baseMean	log_2_ fold change	lfcSE	*p*adj
								
Actinobacteria	Actinobacteria	Micrococcales	Microbacteriaceae	*Frondihabitans*	57.97086	−4.96097	1.54935	0.01465
Actinobacteria	Actinobacteria	Micrococcales	Micrococcaceae	*Micrococcus*	97.60023	−4.53600	1.25073	0.00514
Actinobacteria	Actinobacteria	Micrococcales	Micrococcaceae	*Kocuria*	13.95047	−6.69727	1.90552	0.00591
Actinobacteria	Actinobacteria	Micrococcales	Dermabacteraceae	*Brachybacterium*	144.81196	−5.63555	1.58524	0.00553
Actinobacteria	Actinobacteria	Micrococcales	Brevibacteriaceae	*Brevibacterium*	233.08480	−6.05455	1.16893	0.00002
Actinobacteria	Actinobacteria	Corynebacteriales	Nocardiaceae	*Williamsia*	56.78876	−7.79145	2.27969	0.00782
Actinobacteria	Actinobacteria		Dietziaceae	*Dietzia*	33.74808	−10.02806	2.17538	0.00022
Actinobacteria	Thermoleophilia	Solirubrobacterales	Patulibacteraceae	*Patulibacter*	11.39001	−5.57505	1.75776	0.01525
Proteobacteria	Gammaproteobacteria	Legionellales	Coxiellaceae	*Diplorickettsia*	15.20776	−3.41905	1.20995	0.03997
Proteobacteria	Gammaproteobacteria	Enterobacteriales	Enterobacteriaceae	*Serratia*	343.56698	−16.86702	5.17961	0.01297
Proteobacteria	Alphaproteobacteria	Sphingomonadales	Sphingomonadaceae	*Sphingomonas*	736.07041	−2.11996	0.59077	0.00536
Proteobacteria	Alphaproteobacteria	Rickettsiales	Anaplasmataceae	*Wolbachia*	13.85976	−21.25073	5.77625	0.00471
Saccharibacteria	uncultured bacterium	uncultured bacterium	uncultured bacterium	*uncultured bacterium*	40.93864	−7.19182	1.94072	0.00471
Firmicutes	Bacilli	Bacillales	Bacillaceae	*Oceanobacillus*	7.84285	−18.19205	5.80793	0.01643
Firmicutes	Bacilli	Bacillales	Listeriaceae	*Brochothrix*	8.75038	−8.00489	2.57606	0.01688
Firmicutes	Bacilli	Lactobacillales	Carnobacteriaceae	*Desemzia*	8.45595	−12.15891	3.00312	0.00166
Firmicutes	Bacilli	Lactobacillales	Carnobacteriaceae	*Carnobacterium*	1659.98838	−3.66081	0.89935	0.00166
Firmicutes	Bacilli	Bacillales	Staphylococcaceae	*Jeotgalicoccus*	243.77101	−5.78625	1.49281	0.00285
Firmicutes	Clostridia	Clostridiales	Lachnospiraceae	*Tyzzerella 3*	116.39970	−30.00000	5.80680	0.00002

*The faecal microbiome of great tits were analysed using DESeq2 analysis to identify differentially abundant taxa that are associated with fledging success. Significant differences in genus level abundance (adjusted p-value < 0.05) in the faecal microbiota from great tits that are linked to fledging success. The taxonomic classification, the baseMean, the log_2_ fold change, the log fold change Standard Error (lfcSE), and adjusted p-values of the DESeq2 normalised abundance of each genus are shown.*

## Discussion

The taxonomic composition of great tits’ faecal microbial community was characterised by a predominance of the phyla Firmicutes, Proteobacteria, and Actinobacteria, in line with previous studies (kropáčkováet al., 2017; Teyssier et al., 2018a). On genus level, the great tit microbiome was dominated by *Staphylococcus*, *Bacillus*, *Lactobacillus*, *Carnobacterium*, and *Burkholderia-Paraburkholderia* which corresponds with previous research (Goossens et al., 2021). Although some species belonging to these genera (e.g., *Staphylococcus*) can be pathogenic in birds, these most common genera contain a multitude of beneficial bacteria (Benskin et al., 2009; Grond et al., 2018). We showed that when taking both tree species and edge density into account, a significant effect on alpha and beta diversity was observed, but with a direction and intensity depending on the local tree species richness or tree species composition. Wild birds and their habitat are inextricably linked to human activities as their natural environment is subjected to an increasing pressure of fragmentation. Despite relevant proof in mammals (Amato et al., 2013; Fackelmann et al., 2021) and data indicating that habitat type can influence the microbiome of certain birds (San Juan et al., 2020), it remains largely unknown whether the gut microbiomes of passerines are affected by habitat fragmentation and/or whether other factors are also involved. Our data now show that combined changes in forest structure also have the potential to shift gut microbial communities in passerines.

Microbial recruitment to young bird guts may occur through various routes. Parental effects are the most likely explanation for the patterns observed. Although parents feed insect prey directly, without ingestion and regurgitation, parents can influence their offspring’s gut microbiota through saliva transfer or through variable prey selection (Pagani-Núñez et al., 2015). In forest fragments, the abundance and diversity of arthropods is higher at edges (e.g., De Smedt et al., 2019; van Schrojenstein Lantman et al., 2019). Changes in food resources for great tits could thus explain the changes in the great tit microbiome in habitats having a higher edge density. Possibly, in monocultures which have a resource-poor status (Yahya et al., 2017), more forest edges and thus more insect preys, results in an increase in OTU richness (Chao1), whereas in mixed forest stands this is not the case due to a higher biodiversity. These data are in line with previously published work where a positive edge effect was described on arthropod abundance in monocultures, whereas this relationship was negated in habitats having a higher tree diversity (van Schrojenstein Lantman et al., 2019). With about 29% of Europe’s forests only being composed of a single tree species (Barsoum et al., 2016) and great tits being widespread and common forest birds, our data highlight that changes in the landscape may affect the gut microbiome of wild birds.

Due to the small sample size in this study, we were not able to reliably determine the edge effect per tree species. However, abundance, richness, and diversity of insects may differ greatly depending on the tree identity. Native *Q. robur* is characterised by a species-rich arthropod community (Southwood et al., 2004) and in native *F. sylvatica* monocultures a higher arthropod abundance has been described closer to the edges (van Schrojenstein Lantman et al., 2019). *Q. rubra* is an invasive species in Belgium, and only supports a low abundance and diversity of herbivorous insects (Goßner, 2004). This species forms a dense shrub layer, especially if light is available (Dey and Parker, 1996). It is therefore very likely that in monocultures, edge density influences the microbial diversity differently depending on the local tree species type and that changes in food resources are linked to the observed shifts in the microbiome (Bodawatta et al., 2021).

Besides habitat-driven dietary changes, the observed patterns could also be linked to differences in nest material or even hatching date. Nest material can shape the bacterial community in the nest, which in turn can colonise the gut of nestlings (van Veelen et al., 2017). In great tits, nest material composition, weight and size have previously been found to vary with local tree species composition (Alvarez et al., 2013). Tit nests can also contain anthropogenic material and hairs of domestic animals (Reynolds et al., 2019), which could be more common in small forest fragments or at edges between forests and residential areas. Thus, potentially tree species composition and forest edges can jointly shape material and thus the bacterial community in the nest. Differences in hatching date could possibly also influence the great tit microbiome (Kreisinger et al., 2018). In our study, Julian dates ranged from 120 to 131 (Supplementary Table 3) and did not significantly impact the alpha (LM *p*-values > 0.05), nor the beta diversity (PERMANOVA *p*-values > 0.05). However, previous studies have shown that Julian date can be a confounding factor influencing the microbiome in passerines (Kreisinger et al., 2018).

When looking at abundance of genera, DESeq2 analysis showed that monoculture plots characterised by more edges show a decrease in *Lactococcus*. This genus comprises gram-positive lactic acid bacteria that act as probiotics, stimulate the immune system and aid in the digestion and absorption of nutrients (Salminen et al., 2004). A significant increase of *Pseudarthrobacter*, *Arthrobacter*, and *Erysipelatoclostridium* was observed in monoculture plots having more edges. The genus *Arthrobacter* has previously been linked to diet changes in great tits (Davidson et al., 2020) and the genus *Pseudarthrobacter* was shown to be increased in pine monocultures in which thinning practices led to the formation of a native understory vegetation (Trentini et al., 2020). As such, the increased abundance of *Pseudarthrobacter* is possibly linked to more solar radiation at the edges which results in more favourable conditions for the underlying forest layers. The genus *Erysipelatoclostridium* belongs to the normal gut microbiome of birds (Zhao et al., 2019), but it is also considered an opportunistic pathogen in humans (Shao et al., 2017).

The exact impact of these microbial changes or how gut microbiota affect the health of wild birds in general remains largely unknown (Grond et al., 2018). In great tit nestlings, a higher diversity and stability in microbiota composition were associated with better bird condition (Teyssier et al., 2018a), whereas in house sparrows, hosting a diverse and abundant microbiota flora limits the growth of developing nestlings (Kohl et al., 2018). In our study, the alpha and beta diversity did not significantly impact fledging success of the great tits on nest level. However, other than the lack of an overall microbiome effect, specific bacterial genera were shown to be linked to the fledging success. *Tyzzerella 3* showed a fold change (Log_2_) of -30, indicating that this genus is strongly associated with a reduction in fledging success. *Tyzzerella 3* has previously been linked to diabetes (Yue et al., 2019; Ma et al., 2020), cardiovascular diseases (Kelly et al., 2016) and even reduced disease resistance in poultry (Cazals et al., 2022). The abundance of *Brachybacterium*, *Brevibacterium*, *Serratia*, and *Sphingomonas*, was also negatively linked to fledging success. In general, these genera are not described as common avian pathogenic genera, but they comprise known pathogenic species in many animals, including humans and birds (Pascual and Collins, 1999; Saidenberg et al., 2007; Saeb et al., 2014; Tamai et al., 2018). These data hint towards an adverse health effect of specific bacterial genera, rather than a major effect of the overall microbial richness and diversity. In this study, we did not observe an effect of the microbial changes on the average fledgling SMI per nestbox. However, since we only analysed a short-term effect of the microbial changes on the body condition of the great tits, we cannot rule out whether the observed microbial changes bare no consequences on the long-term. Therefore, further studies are needed to identify the long-term effects of the environmental-driven changes in alpha and beta diversity on host characteristics of great tits. Summarised, we showed that more habitat edges in combination with changes in tree species diversity can influence the microbial richness and phylogenetic diversity in great tits during a life stage where the birds’ microbiota is shaped, which can lead to long-term consequences for host fitness.

## Data Availability Statement

The datasets presented in this study can be found in online repositories. The names of the repository/repositories and accession number(s) can be found below: https://www.ncbi.nlm.nih.gov/, PRJNA615317.

## Ethics Statement

The animal study was reviewed and approved by Ethical Committee VIB (the Flanders Institute for Biotechnology) Ghent site (EC2015-023).

## Author Contributions

AM, LL, KV, DB, FP, DD, and EV conceived the study and participated in its design and coordination. RB and DD performed material preparation and sample collection. SVP, EG, and EV performed the sample analysis. EG, EV, and LH performed the statistical analysis. EV wrote the first draft of the manuscript. All authors commented on previous versions of the manuscript, and read and approved the final manuscript.

## Conflict of Interest

The authors declare that the research was conducted in the absence of any commercial or financial relationships that could be construed as a potential conflict of interest.

## Publisher’s Note

All claims expressed in this article are solely those of the authors and do not necessarily represent those of their affiliated organizations, or those of the publisher, the editors and the reviewers. Any product that may be evaluated in this article, or claim that may be made by its manufacturer, is not guaranteed or endorsed by the publisher.

## Footnotes

1
http://www.bioinformatics.babraham.ac.uk/projects/fastqc/

## References

[B1] Alvarez E. Belda E. J. Verdejo J. Barba E. (2013). Variation in Great Tit Nest Mass and Composition and Its Breeding Consequences: a Comparative Study in four Mediterranean Habitats. *Avian Biol. Res.* 6 39–46. 10.3184/175815513X13609517587237

[B2] Amato K. R. Yeoman C. Kent A. Righini N. Carbonero F. Alejandro E. (2013). Habitat degradation impacts black howler monkey (*Alouatta pigra*) gastrointestinal microbiomes. *ISME J.* 7 1344–1353. 10.1038/ismej.2013.16 23486247 PMC3695285

[B3] Barsoum N. Coote L. Eycott A. E. Fuller L. Kiewitt A. Davies R. G. (2016). Diversity, functional structure and functional redundancy of woodland plant communities: how do mixed tree species plantations compare with monocultures? *For. Ecol. Manag.* 382 244–256. 10.1016/j.foreco.2016.10.005

[B4] Benskin C. M. H. Wilson K. Jones K. Hartley I. A. (2009). Bacterial pathogens in wild birds: a review of the frequency and effects of infection. *Biol. Rev.* 84 349–373. 10.1111/j.1469-185X.2008.00076.x 19438430

[B5] Bodawatta K. H. Freiberga I. Puzejova K. Sam K. Poulsen M. Jønsson K. A. (2021). Flexibility and resilience of great tit (*Parus major*) gut microbiomes to changing diets. *Anim. Microgiome* 3:20. 10.1186/s42523-021-00076-6 33602335 PMC7893775

[B6] Bokulich N. A. Subramanian S. Faith J. J. Gevers D. Gordon J. I. Knight R. (2013). Quality-filtering vastly improves diversity estimates from illumine amplicon sequencing. *Nat. Methods* 10 57–59. 10.1038/nmeth.2276 23202435 PMC3531572

[B7] Bolger A. M. Lohse M. Usadel B. (2014). Trimmomtic: a flexible trimmer for illumina sequence data. *Bioinformatics* 30 2114–2120. 10.1093/bioinformatics/btu170 24695404 PMC4103590

[B8] Bueno-Enciso J. Ferrer E. S. Barrientos R. Serrano-Davies E. Sanz J. J. (2016). Habitat fragmentation influences nestling growth in Mediterranean blue and great tits. *Acta Oecol.* 70 129–137. 10.1016/j.actao.2015.12.008

[B9] Burke D. M. Nol E. (1998). Influence of Food Abundance, Nest-Site Habitat, and forest fragmentation on breeding ovenbirds. *Auk* 115 96–104. 10.2307/4089115

[B10] Cazals A. Estellé J. Bruneau N. Coville J.-L. Menanteau P. Rossignol M.-N. (2022). Differences in caecal microbiota composition and *Salmonella* carriage between experimentally infected inbred lines of chickens. *Genet. Sel. Evol.* 54:7. 10.1186/s12711-022-00699-6 35093028 PMC8801081

[B11] Cenit M. C. Sanz Y. Codoñer-Franch P. (2017). Influence of gut microbiota on neuropsychiatric disorders. *World J. Gastroenterol.* 23 5486–5498. 10.3748/wjg.v23.i30.5486 28852308 PMC5558112

[B12] Davidson G. L. Wiley N. Cooke A. C. Johnson C. N. Fouhy F. Reichert M. S. (2020). Diet induces parallel changes to the gut microbiota and problem solving performance in a wild bird. *Sci. Rep.* 10:20783. 10.1038/s41598-020-77256-y 33247162 PMC7699645

[B13] De Groote S. R. van Schrojenstein L. Irene M. Sercu B. K. Dekeukeleire D. Boonyarittichaikij R. (2017). Tree species identity outweighs the effects of tree species diversity and forest fragmentation on understorey diversity and composition. *Plant Ecol. Evol.* 150 229–239. 10.5091/plecevo.2017.1331

[B14] De Smedt P. Baeten L. Proesmans W. Van de Poel S. Van Keer J. Giffard B. (2019). Strength of forest edge effects on litter-dwelling macro-arthropods across Europe is influenced by forest age and edge properties. *Diver. Distrib.* 25 963–974. 10.1111/ddi.12909

[B15] Dekeukeleire D. Hertzog L. Vantieghem P. van Schrojenstein lantman I. Sercu B. Boonyarittichaikij R. (2019). Forest fragmentation and tree species composition jointly shape breeding performance of two avian insectivores. *For. Ecol. Manag.* 443 95–105. 10.1016/j.foreco.2019.04.023

[B16] Dey D. C. Parker W. C. (1996). Regeneration of red oak (*Quercus rubra L*.) using shelterwood systems: ecophysiology, silviculture and management recommendations. *For. Res. Inf. Pap.* 126 1–59.

[B17] Ding J. Dai R. Yang L. He C. Xu K. Liu S. (2017). Inheritance and establishment of gut microbiota in chickens. *Front. Microbiol.* 8:1967. 10.3389/fmicb.2017.01967 29067020 PMC5641346

[B18] Dixon F. (2003). VEGAN, a package of R functions for community ecology. *J. Veg. Sci.* 14 927–930. 10.1111/j.1654-1103.2003.tb02228.x

[B19] Edgar R. C. (2010). Search and clustering orders of magnitude faster than BLAST. *Bioinformatics* 26 2460–2461. 10.1093/bioinformatics/btq461 20709691

[B20] Edgar R. C. Haas B. J. Clemente J. C. Quince C. Knight R. (2011). UCHIME improves sensitivity and speed of chimera detection. *Bioinformatics* 27 2194–2200. 10.1093/bioinformatics/btr381 21700674 PMC3150044

[B21] Ewers R. M. Thorpe S. Didham R. K. (2007). Synergistic Interactions Between Edge and Area Effect In a Heavily Fragmented Landscape. *Ecology* 88 96–106. 10.1890/0012-9658(2007)88[96:sibeaa]2.0.co;2 17489458

[B22] Fackelmann G. Gillingham M. A. F. Schmid J. Heni A. C. Wilhelm K. Schwensow N. (2021). Human encroachment into wildlife gut microbiomes. *Commun. Biol.* 4:800. 10.1038/s42003-021-02315-7 34172822 PMC8233340

[B23] Fisher J. Lindenmayer D. B. (2007). Landscape modification and habitat fragmentation: a synthesis. *Glob. Ecol. Biogeogr.* 16 265–280. 10.1111/j.1466-8238.2007.00287.x

[B24] Fuentes-Montemayor E. Goulson D. Cavin L. Wallace J. M. Park K. J. (2012). Factors influencing moth assemblages in woodland fragments on farmland: implications for woodland management and creation schemes. *Biol. Conserv.* 153 265–275. 10.1016/j.biocon.2012.04.019

[B25] Goossens E. Boonyarittichaikij R. Dekeukeleire D. Van Praet S. Bonte D. Verheyen K. (2021). Exploring the faecal microbiome of the Eurasian nuthatch (*Sitta europaea*). *Arch. Microbiol.* 203 2119–2127. 10.1007/s00203-021-02195-9 33606040

[B26] Goßner M. (2004). *Diversität und Struktur arborikoler Arthropoden zönosen fremdländischer und einheimischer Baumarten.* Ph.D. thesis. Germany: Technische Universität München.

[B27] Griffiths R. I. Whiteley A. S. O’Donnell A. G. Bailey M. J. (2000). Rapid method for coextraction of DNA and RNA from natural environments for analysis of ribosomal DNA- and rRNA-based microbial community composition. *Appl. Environ. Microbiol.* 66 5488–5491. 10.1128/aem.66.12.5488-5491.2000 11097934 PMC92488

[B28] Grizotte-Lake M. Zhong G. Duncan K. Kirkwood J. Iyer N. Smolenski I. (2018). Commensals suppress intestinal epithelial cell retinoic acid synthesis to regulate interleukin-22 activity and prevent microbial dysbiosis. *Immunity* 49 1103–1115. 10.1016/j.immuni.2018.11.018 30566883 PMC6319961

[B29] Grond K. Sandercock B. K. Jumpponen A. Zeglin L. H. (2018). The avian gut microbiota: community, physiology and function in wild birds. *J. Avian Biol.* 49:e01788. 10.1111/jav.01788

[B30] Hertzog L. R. (2018). *addSE: an R package to add standard errors. Github repository.* Available Online at: https://github.com/lionel68/addSE (accessed September 24, 2021).

[B31] Hertzog L. R. Boonyarittichaikij R. Dekeukeleire D. De Groote S. R. E. Van Schrojenstein Lantman I. M. Sercu B. K. (2019). Forest fragmentation modulates effects of tree species richness and composition on ecosystem multifunctionality. *Ecology* 100:e02653. 10.1002/ecy.2653 30870588

[B32] Hird S. M. Carstens B. C. Cardiff S. W. Dittmann D. L. Brumfield R. T. (2014). Sampling locality is more detectable than taxonomy or ecology in the gut microbiota of the brood-parasitic brown-headed cowbird (*Molothrus ater*). *PeerJ* 2:e321. 10.7717/peerj.321 24711971 PMC3970801

[B33] Kamada N. Chen G. Y. Inohara N. Núñez G. (2013). Control of pathogens and pathobionts by the gut microbiota. *Nat. Immmunol.* 14 685–690. 10.1038/ni.2608 23778796 PMC4083503

[B34] Kelly T. N. Bazzano L. A. Ajami N. J. He H. Zhao J. Petrosino J. F. (2016). Gut microbiome associates with lifetime cardiovascular disease risk profile among bogalusa heart study participants. *Circ. Res.* 119 956–964. 10.1161/CIRCRESAHA.116.309219 27507222 PMC5045790

[B35] Klindworth A. Pruesse E. Schweer T. Peplies J. Quast C. Horn M. (2013). Evaluation of general 16S ribosomal RNA gene PCR primers for classical and next-generation sequencing-based diversity studies. *Nucleic Acids Res.* 41:e1. 10.1093/nar/gks808 22933715 PMC3592464

[B36] Kohl K. D. Antonio B. Bordenstein S. R. Caviedes-vidal E. Karasov W. H. (2018). Gut microbes limit growth in house sparrow nestlings (*Passer domesticus*) but not through limitations in digestive capacity. *Itegr. Zool.* 13 139–151. 10.1111/1749-4877.12289 29168619 PMC5873389

[B37] Kowalchuk G. A. Naoumenko Z. S. Derikx P. J. L. Felske A. Stephen J. R. Arkhipchenko I. A. (1999). Molecular analysis of ammonia-oxidizing bacteria of the beta subdivision of the class *Proteobacteria* in compost and composted materials. *Appl. Environ. Microbiol.* 65 396–403. 10.1128/AEM.65.2.396-403.1999 9925559 PMC91038

[B38] Kreisinger J. Schmiedová L. Petrželková A. Tomášek O. Adámková M. Michálková R. (2018). Fecal microbiota associated with phytohaemagglutinin-induced immune response in nestlings of a passerine bird. *Ecol. Evol.* 8 9793–9802. 10.1002/ece3.4454 30386575 PMC6202713

[B39] kropáčková K. Pechmanová H. Vinkler M. Svobodová J. Velová H. Těšičký M. (2017). Variation between the oral and faecal microbiota in a free-living passerine bird, the great tit (*Parus major*). *PLoS One* 12:e0179945. 10.1371/journal.pone.0179945 28662106 PMC5491070

[B40] Ma S. You Y. Huang L. Long S. Zhang J. Guo C. (2020). Alterations in gut microbiota of gestational diabetes patients during the first trimester of pregnancy. *Front. Cell. Infect. Microbiol.* 10:58. 10.3389/fcimb.2020.00058 32175285 PMC7056672

[B41] Macke E. Tasiemski A. Massol F. Callens M. Decaestecke E. (2017). Life history and eco-evolutionary dynamics in light of the gut microbiota. *Oikos* 126 508–531. 10.1111/oik.03900

[B42] Macpherson A. J. Harris N. L. (2004). Interactions between commensal intestinal bacteria and the immune system. *Nat. Rev. Immunol.* 4 478–485. 10.1038/nri1373 15173836

[B43] Masella A. P. Bartram A. K. Truszkoski J. M. Brown D. G. Neufeld J. D. (2012). PANDAseq: pAired-eND assembler for illumine sequences. *BMC Bioinformatics* 13:31. 10.1186/1471-2105-13-31 22333067 PMC3471323

[B44] McMurdie P. J. Holmes S. (2013). Phyloseq: an R package for reproducible interactive analysis and graphics of microbiome census data. *PLoS One* 8:e61217. 10.1371/journal.pone.0061217 23630581 PMC3632530

[B45] Monrós J. S. Belda E. J. Emilio B. (2003). Post-fledging survival of individual great tits: the effect of hatching date and fledging mass. *Oikos* 99 481–488. 10.1034/j.1600-0706.2002.11909.x 11841302

[B46] Naef-Daenzer B. (2000). Patch time allocation and patch sampling by foraging great tits and blue tits. *Anim. Behav.* 59 989–999. 10.1006/anbe.1999.1380 10860526

[B47] O’Hara A. M. Shanahan F. (2006). The gut flora as a forgotten organ. *EMBO Rep.* 7 688–693. 10.1038/sj.embor.7400731 16819463 PMC1500832

[B48] Pagani-Núñez E. Valls M. Senar J. C. (2015). Diet specialization in a generalist population: the case of breeding great tits *Parus major* in the Mediterranean area. *Oecologia* 179 629–640. 10.1007/s00442-015-3334-2 25983114

[B49] Pascual C. Collins M. D. (1999). *Brevibacterium avium* sp. *nov*., isolated from poultry. *Int. J. Syst. Bacteriol.* 49 1527–1530. 10.1099/00207713-49-4-1527 10555333

[B50] Peig J. Green A. J. (2009). New perspectives for estimating body condition from mass/length data: the scaled mass index as an alternative method. *Oikos* 118 1883–1891. 10.1111/j.1600-0706.2009.17643.x

[B51] Quast C. Pruesse E. Yilmaz P. Gerken J. Schweer T. Yarza P. (2013). The SILVA ribosomal RNA gene database project: improved data processing and web-based tools. *Nucleic Acids Res.* 41 D590–D596. 10.1093/nar/gks1219 23193283 PMC3531112

[B52] R Core Team (2018). *R: a language and environment for statistical computing.* Vienna, Austria: R Foundation for Statistical Computing.

[B53] Reynolds S. J. Ibáñez-Álamo J. D. Sumasguther P. Mainwaring M. C. (2019). Urbanisation and nest building in birds: a review of threats and opportunities. *J. Ornithol.* 160 841–860. 10.1007/s10336-021-01884-y

[B54] Rodríguez S. van Noordwijk A. J. Álvarez E. Barba E. (2016). A recipe for postfledging survival in great tits *Parus major*: be large and be early (but not too much). *Ecol. Evol.* 13 4458–4467. 10.1002/ece3.2192 27386088 PMC4930993

[B55] Saeb A. T. M. David S. K. Al-Brahim H. (2014). In silico detection of virulence gene homologues in the human pathogen *Sphingomonas* spp. *Evol. Bioinform. Online* 10 229–238. 10.4137/EBO.S20710 25574122 PMC4266192

[B56] Saidenberg A. B. S. Teixeira R. H. F. Astolfi-Ferreira C. S. Knöbl T. Ferreira A. J. P. (2007). *Serratia marcescens* infection in a swallow-tailed hummingbird. *J. Wildl. Dis.* 43 107–110. 10.7589/0090-3558-43.1.107 17347399

[B57] Salminen S. Wright A. V. Ouwehand A. C. (2004). *Latic acid bacteria: microbiology and functional aspects.* New York: CRC press.

[B58] San Juan P. A. Hendershot J. N. Daily G. C. Fukami T. (2020). Land-use change has host-specific influences on avian gut microbiomes. *ISME J.* 14 318–321. 10.1038/s41396-019-0535-4 31624349 PMC6908588

[B59] Shao T. J. Shao L. Li H. C. Xie Z. J. He Z. X. Wen C. P. (2017). Combined signature of the fecal microbiome and metabolome in patient with gout. *Front. Microbiol.* 8:268. 10.3389/fmicb.2017.00268 28270806 PMC5318445

[B60] Shutt J. Bolton M. Cabello I. B. Burgess M. D. Phillimore A. (2018). The effects of woodland habitat and biogeography on blue tit *Cyanistes caeruleus* territory occupancy and productivity along a 220 km transect. *Ecography* 41 1967–1978. 10.1111/ecog.03573

[B61] Southwood T. R. E. Wint G. R. W. Kennedy C. E. J. Greenwood S. R. (2004). Seasonality, abundance, species richness and specificity of the phytophagous guild of insects on oak (*Quercus*) canopies. *Eur. J. Entomol.* 101 43–50.

[B62] Tamai K. Akashi Y. Yoshimoto Y. Yaguchi Y. Takeuchi Y. Shiiai M. (2018). First case of a bloodstream infection caused by the genus *Brachybacterium*. *J. Infect. Chemother.* 24 998–1003. 10.1016/j.jiac.2018.06.005 30007866

[B63] Teyssier A. Lens L. Matthysen E. White J. (2018a). Dynamics of gut microbiota during the early development of an avian host: evidence from a cross-foster experiment. *Front. Microbiol.* 9:1524. 10.3389/fmicb.2018.01524 30038608 PMC6046450

[B64] Teyssier A. Rouffaer L. O. Hudin N. S. Strubbe D. Matthysen E. Lens L. (2018b). Inside the guts of the city: urban-induced alterations of the gut microbiota in a wild passerine. *Sci. Total Environ.* 612 1276–1286. 10.1016/j.scitotenv.2017.09.035 28898933

[B65] Trentini C. P. Campanello P. I. Villagra M. Ferreras J. Hartmann M. (2020). Thinning Partially Mitigates the Impact of Atlantic Forest Replacement by Pine Monocultures on the Soil Microbiome. *Front. Microbiol.* 11:1491. 10.3389/fmicb.2020.01491 32719665 PMC7350009

[B66] van Schrojenstein Lantman I. M. Hertzog L. R. Vandegehuchte M. L. Martel A. Verheyen K. Lens L. (2019). Forest edges, tree diversity and tree identity change leaf miner diversity in a temperate forest. *Insect Conserv. Divers.* 13 10–22. 10.1111/icad.12358

[B67] van Veelen H. P. J. Salles J. D. Tieleman B. I. (2017). Multi-level comparisons of cloacal, skin, feather and nest-associated microbiota suggest considerable influence of horizontal acquisition on the microbiota assembly of sympatric woodlarks and skylarks. *Microbiome* 5:156. 10.1186/s40168-017-0371-6 29191217 PMC5709917

[B68] Vriens L. Bosch H. De Knijf G. De Saeger S. Guelinckx R. Oosterlynck P. (2011). *De biologische waarderingskaart. Biotopen en hun verspreiding in Vlaanderen en het Brussels Hoofdstedelijk Gewest. Mededelingen van het Instituut voor Natuur- en Bosonderzoek. INBO.M. 1.* Brussel: Mededelingen van het Instituut voor Natuur- en Bosonderzoek, 416.

[B69] Yahya M. S. Syafiq M. Ashton-Butt A. Ghazali A. Asmah S. Azhar B. (2017). Switching from monoculture to polyculture farming benefits birds in oil palm production landscapes: evidence from mist netting data. *Ecol. Evol.* 16 6314–6325. 10.1002/ece3.3205 28861235 PMC5574735

[B70] Yue S. Zhao D. Peng C. Tan C. Wang Q. Gong J. (2019). Effects of theabrownin on serum metabolites and gut microbiome in rats with a high-sugar diet. *Food Funct.* 10 7063–7080. 10.1039/C9FO01334B 31621728

[B71] Zanette L. Doyle P. Trémont S. M. (2000). Food shortage in small fragments: evidence from an area-sensitive passerine. *Ecology* 81 1654–1666. 10.2307/177314

[B72] Zhao Y. Li K. Luo H. Duan L. Wei C. Wang M. (2019). Comparison of the intestinal microbial community in duck reared differently through high-throughput sequencing. *Biomed Res. Int.* 2019 1–14. 10.1155/2019/9015054 30956988 PMC6431443

[B73] Zhu X. Han Y. Du J. Liu R. Jin K. Yi W. (2017). Microbiota-gut-brain axis and the central nervous system. *Oncotarget* 8 53829–53838. 10.18632/oncotarget.17754 28881854 PMC5581153

