# Supplementary material for: Tree Species Diversity and Forest Edge Density Jointly Shape the Gut Microbiota Composition in Juvenile Great Tits (Parus major)
Source: Front Microbiol. 2022 Mar 9;13:790189. doi: 10.3389/fmicb.2022.790189 (PMC8959704; doi:10.3389/fmicb.2022.790189)
Supplement: Supplementary file 2 [file Data_Sheet_2.docx]

**Tree species diversity and forest edge density jointly shape the gut microbiota composition in juvenile great tits (*Parus major*)**

Running title: Faecal microbiota of great tits

Evy Goossens^1,†^, Roschong Boonyarittichaikij^1,2,†^, Daan Dekeukeleire^3^, Lionel Hertzog^3^, Sarah Van Praet^1^, Frank Pasmans^1^, Dries Bonte^3^, Kris Verheyen^4^, Luc Lens^3^, An Martel^1,§^, Elin Verbrugghe^1,§,*^

**Supplementary Material**

**
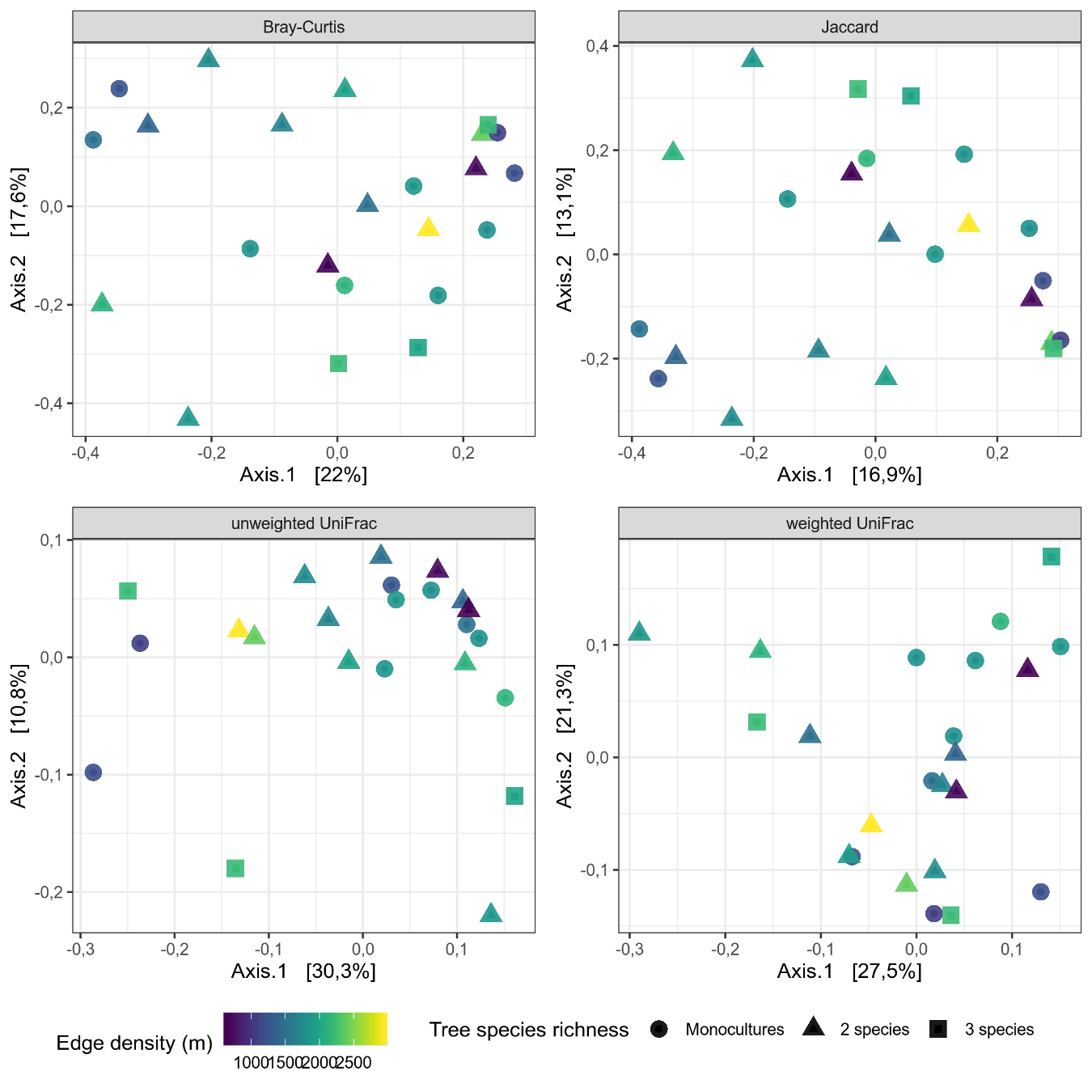
**

**Supplementary Figure S1:** A principle coordinate analysis (PCoA) plot was created for four dissimilarity indexes: Bray-Curtis, Jaccard, unweighted UniFrac and weighed UniFrac. Dots, triangles and squares represent monocultures, 2 species plots and 3 species plots, respectively, along a gradient of edge density, as represented by a color code.

**Supplementary Table S1: Overview of the tree diversity per plot.**

| **Longitude** | **Latitude** | **Plot number** | **Number of sampled nestlings** | **Tree**  **species richness** | **Tree**  **species composition** |
| --- | --- | --- | --- | --- | --- |
| 3.7737 | 50.9905 | 1 | 1 | 2 | qrob_qrub |
| 3.7353 | 50.9610 | 2 | 3 | 1 | fsyl |
| 3.7326 | 50.9602 | 3 | 1 | 1 | fsyl |
| 3.7282 | 50.9485 | 4 | 1 | 2 | fsyl_qrub |
| 3.7290 | 50.9488 | 5 | 2 | 3 | all |
| 3.7178 | 50.9461 | 12 | 3 | 2 | fsyl_qrub |
| 3.7122 | 50.9440 | 18 | 5 | 1 | qrob |
| 3.7603 | 50.9169 | 25 | 2 | 2 | qrob_qrub |
| 3.8711 | 50.9110 | 28 | 3 | 2 | fsyl_qrob |
| 3.8719 | 50.9136 | 29 | 1 | 3 | all |
| 3.9475 | 50.9849 | 33 | 1 | 1 | qrub |
| 3.9487 | 50.9821 | 35 | 2 | 1 | qrob |
| 3.9294 | 50.9768 | 37 | 1 | 1 | qrub |
| 3.9067 | 50.9763 | 39 | 4 | 2 | fsyl_qrub |
| 3.9089 | 50.9756 | 41 | 2 | 1 | qrub |
| 3.9067 | 50.9729 | 42 | 5 | 1 | fsyl |
| 3.9077 | 50.9702 | 43 | 1 | 1 | qrob |
| 3.9078 | 50.9713 | 44 | 4 | 2 | fsyl_qrob |
| 3.8383 | 50.9623 | 49 | 2 | 2 | qrob_qrub |
| 3.8405 | 50.9640 | 51 | 2 | 2 | qrob_qrub |
| 3.5884 | 50.9961 | 52 | 1 | 2 | qrob_qrub |
| 3.5852 | 50.9975 | 53 | 2 | 2 | qrob_qrub |

Qrub= *Quercus rubra*; fsyl = *Fagus sylvatica*; qrob= *Quercus robur*

**Supplementary Table S2: Overview of the fragmentation metrics.**

| **plot** | **Surface area** | **shape** | **prox** | **Nearest**  **neighb** | **Edge**  **length** | **Edge**  **density** | **Forest**  **100m** | **Forest**  **300m** | **Forest**  **500m** | **Edge**  **100m** | **Edge**  **300m** | **Closest**  **edge** |
| --- | --- | --- | --- | --- | --- | --- | --- | --- | --- | --- | --- | --- |
| 1 | 25.36 | 2.41 | 1.05 | 286.40 | 4862.71 | 191.77 | 1.73 | 13.44 | 23.22 | 423.87 | 2932.40 | 12.94 |
| 2 | 43.74 | 1.85 | 44.57 | 177.20 | 4898.68 | 112.00 | 2.48 | 14.80 | 25.24 | 176.06 | 1811.17 | 46.84 |
| 3 | 43.74 | 1.85 | 44.57 | 177.20 | 4898.68 | 112.00 | 2.56 | 18.24 | 30.24 | 175.28 | 1202.99 | 54.91 |
| 4 | 29.83 | 1.83 | 69.31 | 20.62 | 4000.32 | 134.13 | 3.14 | 17.40 | 32.40 | 0.00 | 2392.47 | 130.63 |
| 5 | 29.83 | 1.83 | 69.31 | 20.62 | 4000.32 | 134.13 | 3.08 | 15.20 | 29.76 | 86.96 | 2200.02 | 90.06 |
| 12 | 90.36 | 2.45 | 82.03 | 53.85 | 9332.29 | 103.28 | 2.88 | 22.11 | 47.35 | 179.23 | 1747.12 | 53.09 |
| 18 | 90.36 | 2.45 | 82.03 | 53.85 | 9332.29 | 103.28 | 2.02 | 12.76 | 28.12 | 401.43 | 1258.83 | 43.14 |
| 25 | 19.30 | 1.22 | 1.43 | 219.20 | 2140.57 | 110.92 | 1.99 | 10.51 | 15.64 | 275.70 | 1495.38 | 96.32 |
| 28 | 10.74 | 2.01 | 57.99 | 30.41 | 2635.52 | 245.33 | 1.55 | 7.83 | 11.59 | 419.10 | 1861.98 | 45.19 |
| 29 | 10.74 | 2.01 | 57.99 | 30.41 | 2635.52 | 245.33 | 2.36 | 6.77 | 12.48 | 312.28 | 1982.89 | 25.90 |
| 33 | 35.49 | 2.38 | 10.09 | 15.81 | 5671.47 | 159.80 | 2.03 | 15.62 | 30.30 | 194.57 | 1787.88 | 23.34 |
| 35 | 35.49 | 2.38 | 10.09 | 15.81 | 5671.47 | 159.80 | 3.13 | 17.97 | 27.34 | 28.94 | 2150.79 | 88.96 |
| 37 | 58.35 | 3.58 | 14.19 | 49.24 | 10928.77 | 187.29 | 3.14 | 15.60 | 30.60 | 0.00 | 1834.15 | 106.25 |
| 39 | 46.64 | 2.12 | 53.59 | 30.00 | 5793.11 | 124.20 | 3.14 | 22.51 | 34.02 | 0.00 | 2118.24 | 171.65 |
| 41 | 46.64 | 2.12 | 53.59 | 30.00 | 5793.11 | 124.20 | 3.13 | 20.57 | 34.17 | 18.55 | 1874.24 | 93.18 |
| 42 | 46.64 | 2.12 | 53.59 | 30.00 | 5793.11 | 124.20 | 2.68 | 20.50 | 38.51 | 161.74 | 1505.20 | 59.14 |
| 43 | 46.64 | 2.12 | 53.59 | 30.00 | 5793.11 | 124.20 | 2.14 | 13.59 | 22.12 | 188.38 | 1077.37 | 25.93 |
| 44 | 46.64 | 2.12 | 53.59 | 30.00 | 5793.11 | 124.20 | 3.12 | 17.57 | 27.60 | 39.20 | 1378.85 | 89.95 |
| 49 | 25.50 | 1.49 | 2.04 | 301.87 | 3001.75 | 117.71 | 3.05 | 21.73 | 34.20 | 78.44 | 1946.66 | 69.80 |
| 51 | 25.50 | 1.49 | 2.04 | 301.87 | 3001.75 | 117.71 | 2.99 | 14.43 | 30.85 | 103.62 | 1674.36 | 85.59 |
| 52 | 53.39 | 1.39 | 8.18 | 60.00 | 4069.36 | 76.22 | 3.14 | 22.56 | 40.03 | 0.00 | 703.80 | 181.23 |
| 53 | 53.39 | 1.39 | 8.18 | 60.00 | 4069.36 | 76.22 | 3.14 | 26.46 | 45.15 | 0.00 | 655.91 | 199.37 |

See Dekeukeleire et al. (2019) for more information on the fragmentation variables.

**Supplementary Table S3: Overview of the SMI of the sampled birds and the fledging success per nest.**

| **Bird ring number** | **Accession number** | **plot N°** | **Nestbox number** | **SMI** | **Average SMI per nestbox** | **N° nestlings** | **N° fledglings** | **Fledging success per nestbox** | **Hatching date: Julian Date** |
| --- | --- | --- | --- | --- | --- | --- | --- | --- | --- |
| 58V 929 37 | SAMN14450675 | 1 | 1_4 | NC | NC | 8 | 8 | 1.00 | 122 |
| 58V 928 53 | SAMN14450641 | 2 | 2_3 | 18.67 | 17.85 | 5 | 5 | 1.00 | 131 |
| 58V 928 54 | SAMN14450642 | 2 | 2_3 | 17.55 |  | 5 | 5 |  | 131 |
| 58V 928 55 | SAMN14450643 | 2 | 2_3 | 17.33 |  | 5 | 5 |  | 131 |
| 58V 928 64 | SAMN14450644 | 3 | 3_4 | 16.99 | 16.99 | 7 | 4 | 0.57 | 131 |
| 58V 928 15 | SAMN14450635 | 4 | 4_1 | 19.84 | 19.84 | 5 | 5 | 1.00 | 124 |
| 58V 927 96 | SAMN14450633 | 5 | 5_1 | 16.91 | 16.91 | 2 | 2 | 1.00 | 129 |
| 58V 927 99 | SAMN14450634 | 5 | 5_2 | 16.23 | 16.23 | 4 | 4 | 1.00 | 129 |
| 58V 928 66 | SAMN14450645 | 12 | 12_3 | 18.22 | 18.02 | 7 | 7 | 1.00 | 131 |
| 58V 928 68 | SAMN14450646 | 12 | 12_3 | 16.90 |  | 7 | 7 |  | 131 |
| 58V 928 69 | SAMN14450647 | 12 | 12_3 | 18.95 |  | 7 | 7 |  | 131 |
| 58V 928 73 | SAMN14450648 | 18 | 18_1 | 17.76 | 19.14 | 10 | 10 | 1.00 | 131 |
| 58V 928 74 | SAMN14450649 | 18 | 18_1 | 17.11 |  | 10 | 10 |  | 131 |
| 58V 928 75 | SAMN14450650 | 18 | 18_1 | 20.89 |  | 10 | 10 |  | 131 |
| 58V 928 78 | SAMN14450651 | 18 | 18_1 | 20.82 |  | 10 | 10 |  | 131 |
| 58V 928 79 | SAMN14450652 | 18 | 18_1 | 19.14 |  | 10 | 10 |  | 131 |
| 58V 847 97 | SAMN14450623 | 25 | 25_1 | 19.17 | 18.33 | 9 | 8 | 0.89 | 124 |
| 57V 848 00 | SAMN14450624 | 25 | 25_1 | 17.48 |  | 9 | 8 |  | 124 |
| 58V 929 91 | SAMN14450682 | 28 | 28_4 | 19.03 | 18.29 | 6 | 5 | 0.83 | 126 |
| 58V 929 92 | SAMN14450683 | 28 | 28_4 | 17.55 |  | 6 | 5 |  | 126 |
| 58V 929 95 | SAMN14450684 | 28 | 28_4 | NC |  | 6 | 5 |  | 126 |
| 58V 929 98 | SAMN14450685 | 29 | 29_4 | 16.97 | 16.97 | NC | 9 | NC | 126 |
| 58V 928 43 | SAMN14450640 | 33 | 33_1 | 20.92 | 20.92 | 6 | 3 | 0.50 | 125 |
| 58V 929 03 | SAMN14450665 | 35 | 35_3 | NC | NC | 7 | 7 | 1.00 | 120 |
| 58V 929 05 | SAMN14450666 | 35 | 35_3 | NC |  | 7 | 7 |  | 120 |
| 58V 929 00 | SAMN14450664 | 37 | 37_3 | 15.84 | 15.84 | 6 | 3 | 0.50 | 128 |
| 58V 928 83 | SAMN14450653 | 39 | 39_4 | 21.75 | 21.49 | 6 | 0 | 0.00 | 128 |
| 58V 928 84 | SAMN14450654 | 39 | 39_4 | 21.23 |  | 6 | 0 |  | 128 |
| 58V 928 85 | SAMN14450655 | 39 | 39_4 | NC |  | 6 | 0 |  | 128 |
| 58V 928 86 | SAMN14450656 | 39 | 39_4 | NC |  | 6 | 0 |  | 128 |
| 58V 927 87 | SAMN14450631 | 41 | 41_3 | 16.80 | 17.72 | 7 | 6 | 0.86 | 128 |
| 58V 927 89 | SAMN14450632 | 41 | 41_3 | 18.65 |  | 7 | 6 |  | 128 |
| 58V 928 89 | SAMN14450657 | 42 | 42_1 | 19.68 | 17.35 | 6 | 4 | 0.67 | 128 |
| 58V 928 91 | SAMN14450658 | 42 | 42_1 | 17.95 |  | 6 | 4 |  | 128 |
| 58V 928 92 | SAMN14450659 | 42 | 42_1 | 16.68 |  | 6 | 4 |  | 128 |
| 58V 928 94 | SAMN14450661 | 42 | 42_1 | 15.74 |  | 6 | 4 |  | 128 |
| 58V 928 97 | SAMN14450662 | 42 | 42_1 | 16.73 |  | 6 | 4 |  | 128 |
| 58V 928 99 | SAMN14450663 | 43 | 43_2 | 16.73 | 16.73 | 7 | 7 | 1.00 | 128 |
| 58V 927 79 | SAMN14450627 | 44 | 44_1 | 18.13 | 17.43 | 7 | 6 | 0.86 | 128 |
| 58V 927 81 | SAMN14450628 | 44 | 44_1 | 16.74 |  | 7 | 6 |  | 128 |
| 58V 927 82 | SAMN14450629 | 44 | 44_1 | NC |  | 7 | 6 |  | 128 |
| 58V 927 83 | SAMN14450630 | 44 | 44_1 | NC |  | 7 | 6 |  | 128 |
| 58V 928 22 | SAMN14450636 | 49 | 49_1 | 20.28 | 19.33 | 5 | 5 | 1.00 | 125 |
| 58V 928 23 | SAMN14450637 | 49 | 49_1 | 18.38 |  | 5 | 5 |  | 125 |
| 58V 928 27 | SAMN14450638 | 51 | 51_1 | 15.95 | 16.09 | 7 | 7 | 1.00 | 125 |
| 58V 928 33 | SAMN14450639 | 51 | 51_1 | 16.23 |  | 7 | 7 |  | 125 |
| 58V 929 88 | SAMN14450681 | 52 | 52_4 | 18.73 | 18.73 | 8 | NC | NC | 126 |
| 58V 929 84 | SAMN14450679 | 53 | 53_4 | 16.56 | 16.92 | NC | 4 | NC | 126 |
| 58V 929 86 | SAMN14450680 | 53 | 53_4 | 17.29 |  | NC | 4 |  | 126 |

NC: Not collected

**Supplementary Table S4: LM and ANOVA analysis of alpha diversity.** We used linear models and ANOVA (type 1) to analyze the interactions between tree species (composition and richness) and forest fragmentation (fragment area and edge density) on the alpha diversity of faecal microbiota of great tits. The significance of the fragmentation effects for the different tree richness or tree composition levels was assessed using the addSE packages (Hertzog, 2018). Shown are linear models, followed by addSE and ANOVA of the estimated OTU richness (Chao1) and estimated community diversity (Shannon), with significance (p < 0.05) indicated by *. Fsyl = European Beech (*Fagus sylvatica*, L), Qrob = Pedunculate Oak (*Quercus robur*, L), Qrub = Red Oak (*Quercus rubra*, L).

1) Interaction between edge density and tree species composition

| **LM** | **Response: Chao 1** | | **Response: Shannon** | |
| --- | --- | --- | --- | --- |
|  | Coefficient | Pr(>\|t\|) | Coefficient | Pr(>\|t\|) |
| (Intercept) | -261.579 | 0.404 | 0.596 | 0.668 |
| edge density | 0.516 | 0.027* | 0.002 | 0.021* |
| *Fsyl-Qrob* | 1023.915 | 0.072 | 11.535 | 0.001* |
| *Fsyl-Qrob-Qrub* | 1894.780 | 0.106 | 13.384 | 0.020* |
| *Fsyl-Qrub* | 919.635 | 0.093 | 3.564 | 0.140 |
| *Qrob* | 507.364 | 0.171 | 3.211 | 0.066 |
| *Qrob-Qrub* | 788.981 | 0.031* | 4.083 | 0.017* |
| *Qrub* | 956.616 | 0.715 | 40.002 | 0.007* |
| *Fsyl-Qrob*:Edge density | -0.634 | 0.075 | -0.008 | 4.02e-4* |
| *Fsyl-Qrob-Qrub*:Edge density | -1.082 | 0.064 | -0.007 | 0.015* |
| *Fsyl-Qrub*:Edge density | -0.590 | 0.056 | -0.002 | 0.083 |
| *Qrob*:Edge density | -0.354 | 0.145 | -0.002 | 0.079 |
| *Qrob-Qrub*:Edge density | -0.548 | 0.023* | -0.003 | 0.016* |
| *Qrub*:Edge density | -0.620 | 0.667 | -0.022 | 0.006* |

| **Add SE** | **Response: Chao1** | | **Response: Shannon** | |
| --- | --- | --- | --- | --- |
| Explanatory var. | Coefficient | LCI, UCI | Coefficient | LCI, UCI |
| *Fsyl:*Edge density | 0.516 | 0.132, 0.899* | 0.002 | 0.001, 0.004* |
| *Fsyl-Qrob*:Edge density | -0.118 | -0.601, 0.365 | -0.005 | -0.007, -0.003* |
| *Fsyl-Qrob-Qrub*:Edge density | -0.567 | -1.50, 0.364 | -0.004 | -0.009, -2.85e-4* |
| *Fsyl-Qrub*:Edge density | -0.075 | -0.435, 0.286 | 1.00e-4 | -0.002, 0.002 |
| *Qrob*:Edge density | 0.162 | -0.041, 0.365 | 4.77e-4 | -4.38e-4, 0.001 |
| *Qrob-Qrub*:Edge density | -0.032 | -0.119, 0.055 | -2.32e-4 | -0.001, 1.60e-4 |
| *Qrub*:Edge density | -0.104 | -2.804, 2.596 | -0.020 | -0.032, -0.008* |

| **ANOVA** | **Response: Chao1** | | **Response: Shannon** | |
| --- | --- | --- | --- | --- |
| Explanatory var. | F value | Pr(>F) | F value | Pr(>F) |
| Edge density (1, 9) | 0.034 | 0.857 | 0.483 | 0.505 |
| Tree species composition (6,9) | 0.673 | 0.675 | 1.620 | 0.247 |
| Edge density: tree species composition (6,9) | 1.946 | 0.177 | 7.717 | 0.004* |

The parenthesis after the effect names correspond to the numerator and denominator (residual) degrees of freedom.

2) Interaction between edge density and tree species richness

| **LM** | **Response: Chao 1** | | **Response: Shannon** | |
| --- | --- | --- | --- | --- |
| Explanatory var. | Coefficient | Pr(>\|t\|) | Coefficient | Pr(>\|t\|) |
| (Intercept) | 552.986 | 3.54e-07 | 4.665 | 4.52e-07 |
| Edge density | -0.030 | 0.432 | -0.0002 | 0.409 |
| 3 species forest | 1080.216 | 0.276 | 9.316 | 0.274 |
| Monoculture forest | -358.481 | 0.024* | -1.169 | 0.360 |
| 3 species forest:Edge density | -0.536 | 0.251 | -0.004 | 0.293 |
| Monoculture forest:Edge density | 0.221 | 0.019 * | 0.001 | 0.293 |

| **Add SE** | **Response: Chao1** | | **Response: Shannon** | |
| --- | --- | --- | --- | --- |
| Explanatory var. | Coefficient | LCI, UCI | Coefficient | LCI, UCI |
| Monoculture forest:Edge density | 0.191 | 0.040, 0.342* | 0.001 | -0.001, 0.002 |
| 2 species forest:Edge density | -0.030 | -0.104, 0.043 | -2.73e-4 | -0.001, 3.60e-4 |
| 3 species forest:Edge density | -0.567 | -1.448, 0.314 | -0.004 | -0.012, 0.003 |

| **ANOVA** | **Response: Chao1** | | **Response: Shannon** | |
| --- | --- | --- | --- | --- |
| Explanatory var. | F value | Pr(>F) | F value | Pr(>F) |
| Edge density (1, 17) | 0.038 | 0.847 | 0.148 | 0.705 |
| Tree species richness (2, 17) | 1.145 | 0.342 | 0.268 | 0.768 |
| Edge density:tree species richness (2, 17) | 4.157 | 0.034* | 1.223 | 0.319 |

The parenthesis after the effect names correspond to the numerator and denominator (residual) degrees of freedom.

3) Interaction between fragment area and tree species composition

| **Linear model** | **Response: Chao 1** | | **Response: Shannon** | |
| --- | --- | --- | --- | --- |
| Explanatory var. | Coefficient | Pr(>\|t\|) | Coefficient | Pr(>\|t\|) |
| (Intercept) | -2152.78 | 0.329 | 0.820 | 0.945 |
| fragment area | 59.67 | 0.233 | 0.078 | 0.771 |
| *Fsyl-Qrob* | 2677.68 | 0.233 | 0.714 | 0.952 |
| *Fsyl-Qrob-Qrub* | 2731.61 | 0.225 | 4.829 | 0.687 |
| *Fsyl-Qrub* | 2634.14 | 0.240 | 3.580 | 0.765 |
| *Qrob* | 2577.25 | 0.250 | 4.298 | 0.720 |
| *Qrob-Qrub* | 2588.62 | 0.247 | 2.741 | 0.818 |
| *Qrub* | 2741.01 | 0.227 | 3.91 | 0.746 |
| *Fsyl-Qrob*:Fragment area | -58.08 | 0.247 | -0.007 | 0.980 |
| *Fsyl-Qrob-Qrub*:Fragment area | -66.12 | 0.195 | -0.129 | 0.635 |
| *Fsyl-Qrub*:Fragment area | -59.30 | 0.236 | -0.078 | 0.769 |
| *Qrob*:Fragment area | -58.57 | 0.242 | -0.088 | 0.742 |
| *Qrob-Qrub*:Fragment area | -58.45 | 0.243 | -0.055 | 0.836 |
| *Qrub*:Fragment area | -61.47 | 0.225 | -0.089 | 0.742 |

| **Add SE** | **Response: Chao1** | | **Response: Shannon** | |
| --- | --- | --- | --- | --- |
| Explanatory var. | Coefficient | LCI, UCI | Coefficient | LCI, UCI |
| *Fsyl*:Fragment area | 59.674 | -31.789, 151.138 | 0.078 | -0.429, 0.585 |
| *Fsyl-Qrob*:Fragment area | 1.592 | -6.952, 10.137 | 0.071 | 0.024, 0.118* |
| *Fsyl-Qrob-Qrub*:Fragment area | -6.448 | -20.370, 7.474 | -0.051 | -0.128, 0.026 |
| *Fsyl-Qrub*:Fragment area | 0.371 | -4.538, 5.280 | -0.001 | -0.028, 0.027 |
| *Qrob*:Fragment area | 1.103 | -4.186, 6.393 | -0.010 | -0.040, 0.019 |
| *Qrob-Qrub*:Fragment area | 1.224 | -5.072, 7.521 | 0.022 | -0.013, 0.057 |
| *Qrub*:Fragment area | -1.791 | -15.208, 11.626 | -0.011 | -0.086, 0.063 |

| **ANOVA** | **Response: Chao1** | | **Response: Shannon** | |
| --- | --- | --- | --- | --- |
| Explanatory var. | F value | Pr(>F) | F value | Pr(>F) |
| fragment area (1,9) | 0.341 | 0.573 | 1.070 | 0.328 |
| species compostion (6,9) | 0.357 | 0.888 | 0.513 | 0.786 |
| fragment area:species composition (6,9) | 0.474 | 0.812 | 1.979 | 0.172 |

The parenthesis after the effect names correspond to the numerator and denominator (residual) degrees of freedom.

4) Interaction between fragment area and tree species richness

| **LM** | **Response: Chao 1** | | **Response: Shannon** | |
| --- | --- | --- | --- | --- |
| Explanatory var. | Coefficient | Pr(>\|t\|) | Coefficient | Pr(>\|t\|) |
| (Intercept) | 480.934 | 2.62e-07 | 3.504 | 1.02e-07 |
| Fragment area | 0.515 | 0.702 | 0.018 | 0.065 |
| 3 species forest | 97.903 | 0.554 | 2.14 | 0.069 |
| Monoculture forest | -4.654 | 0.969 | 1.150 | 0.17 |
| 3 species forest:Fragment area | -6.963 | 0.274 | -0.069 | 0.119 |
| Monoculture forest:Fragment area | 0.012 | 0.996 | -0.024 | 0.157 |

| **Add SE** | **Response: Chao1** | | **Response: Shannon** | |
| --- | --- | --- | --- | --- |
| Explanatory var. | Coefficient | LCI, UCI | Coefficient | LCI, UCI |
| Fragment area | 0.527 | -3.360, 4.414 | -0.006 | -0.033, 0.020 |
| Species richness | 0.515 | -2.084, 3.114 | 0.018 | 1.31e-04, 0.036* |
| Fragment area:species richness | -6.448 | -18.230, 5.334 | -0.051 | -0.131, 0.029 |

| **ANOVA** | **Response: Chao1** | | **Response: Shannon** | |
| --- | --- | --- | --- | --- |
| Explanatory var. | F value | Pr(>F) | F value | Pr(>F) |
| Monoculture forest:Fragment area (1, 17) | 0.477 | 0.499 | 0.990 | 0.334 |
| 2 species forest:Fragment area (2, 17) | 0.615 | 0.552 | 0.403 | 0.675 |
| 3 species forest:Fragment area (2, 17) | 0.650 | 0.535 | 2.184 | 0.143 |

**Supplementary Table S5: PERMANOVA analysis on beta diversity.** We investigated the influence of tree species (composition and richness) and fragmentation measures (fragment area and edge density) on the beta diversity of faecal microbiota of great tits. Four dissimilarity indexes (Bray-Curtis, Jaccard, weighted UniFrac and unweighted UniFrac) were taken into account. Number of permutations = 999. Significance (p < 0.05) is indicated by *.

| Edge density and tree species composition | Bray-Curtis | | | Jaccard | | | Unweighted UniFrac | | | Weighted UniFrac | | |  |
| --- | --- | --- | --- | --- | --- | --- | --- | --- | --- | --- | --- | --- | --- |
|  | F | R2 | p | F | R2 | p | F | R2 | p | F | R2 | p | |
| Edge density (1, 9) | 0.621 | 0.028 | 0.842 | 0.733 | 0.034 | 0.851 | 0.956 | 0.038 | 0.469 | 1.06 | 0.041 | 0.376 | |
| Tree species composition (6, 9) | 1.00 | 0.274 | 0.485 | 0.956 | 0.265 | 0.603 | 1.026 | 0.244 | 0.451 | 1.056 | 0.242 | 0.395 | |
| Edge density: Tree species composition (6, 9) | 1.052 | 0.287 | 0.443 | 1.030 | 0.286 | 0.419 | 1.526 | 0.362 | 0.041* | 1.631 | 0.374 | 0.055 | |

| Fragment area and tree species composition | Bray-Curtis | | | Jaccard | | | Unweighted UniFrac | | | Weighted UniFrac | | |
| --- | --- | --- | --- | --- | --- | --- | --- | --- | --- | --- | --- | --- |
|  | F | R2 | p | F | R2 | p | F | R2 | p | F | R2 | p |
| Fragment area (1, 9) | 1.443 | 0.065 | 0.137 | 1.305 | 0.060 | 0.128 | 0.772 | 0.038 | 0.683 | 0.701 | 0.034 | 0.704 |
| Species composition (6, 9) | 1.009 | 0.274 | 0.446 | 0.953 | 0.265 | 0.626 | 0.820 | 0.244 | 0.809 | 0.838 | 0.2427 | 0.74 |
| Fragment area: Tree species composition (6, 9) | 0.930 | 0.253 | 0.605 | 0.926 | 0.258 | 0.694 | 0.920 | 0.273 | 0.623 | 1.010 | 0.291 | 0.497 |

| Edge density and tree species richness | Bray-Curtis | | | Jaccard | | | Unweighted UniFrac | | | Weighted UniFrac | | |
| --- | --- | --- | --- | --- | --- | --- | --- | --- | --- | --- | --- | --- |
|  | F | R2 | p | F | R2 | p | F | R2 | p | F | R2 | p |
| Edge density (1, 17) | 0.618 | 0.028 | 0.873 | 0.715 | 0.032 | 0.882 | 1.050 | 0.039 | 0.365 | 0.939 | 0.037 | 0.504 |
| Tree species richness (2, 17) | 0.967 | 0.087 | 0.47 | 0.938 | 0.085 | 0.589 | 1.203 | 0.090 | 0.236 | 1.164 | 0.092 | 0.281 |
| Edge density: Tree species richness (2, 17) | 1.299 | 0.117 | 0.134 | 1.221 | 0.111 | 0.134 | 3.097 | 0.232 | 0.001* | 2.486 | 0.197 | 0.007* |

| Fragment area and tree species richness | Bray-Curtis | | | Jaccard | | | Unweighted UniFrac | | | Weighted UniFrac | | |
| --- | --- | --- | --- | --- | --- | --- | --- | --- | --- | --- | --- | --- |
|  | F | R2 | p | F | R2 | p | F | R2 | p | F | R2 | p |
| Fragment area (1, 17) | 1.637 | 0.072 | 0.071 | 1.395 | 0.063 | 0.078 | 1.003 | 0.045 | 0.39 | 0.951 | 0.040 | 0.454 |
| Tree species richness (2, 17) | 0.991 | 0.087 | 0.451 | 0.949 | 0.085 | 0.56 | 1.013 | 0.090 | 0.397 | 1.101 | 0.092 | 0.327 |
| Fragment area: Tree species richness (2, 17) | 1.038 | 0.091 | 0.392 | 1.004 | 0.090 | 0.476 | 1.211 | 0.108 | 0.268 | 1.857 | 0.156 | 0.057 |

The parenthesis after the effect names correspond to the numerator and denominator (residual) degrees of freedom.

**Supplementary Table S6: Influence of the alpha diversity on host parameters of great tits.** LM output is shown, analyzing the influence of the environmental-driven changes of the alpha diversity on SMI and fledging success. The following alpha diversity metrics were taking into account: estimated OTU richness (Chao1) and estimated community diversity (Shannon).

|  | Response: SMI | |  | Response: SMI | |
| --- | --- | --- | --- | --- | --- |
| Explanatory var. | Coefficient | Pr(>\|t\|) | Explanatory var. | Coefficient | Pr(>\|t\|) |
| (Intercept) | -2.005 | 0.905 | (Intercept) | -0.207 | 0.994 |
| Chao1 | 0.037 | 0.244 | Shannon | 3.123 | 0.596 |
| PUL_NR | 0.585 | 0.709 | PUL_NR | 0.961 | 0.563 |

|  | Response: n° fledglings/n° nestlings | |  | Response: n° fledglings/n° nestlings | |
| --- | --- | --- | --- | --- | --- |
| Explanatory var. | Coefficient | Pr(>\|t\|) | Explanatory var. | Coefficient | Pr(>\|t\|) |
| (Intercept) | 0.066 | 0.927 | (Intercept) | -0.299 | 0.798 |
| Chao1 | -0.001 | 0.728 | Shannon | 0.027 | 0.919 |

**Supplementary Table S7: Influence of the beta diversity on host parameters of great tits.** LM output is shown, analyzing the influence of the environmental-driven changes of the beta diversity on SMI and fledging success. Four dissimilarity indexes (Bray-Curtis, Jaccard, weighted UniFrac and unweighted UniFrac) were taken into account. Number of permutations = 999.

| Bray-Curtis | Response: SMI | | Response: n° fledglings/n° nestlings | |
| --- | --- | --- | --- | --- |
| Explan. var. | Coefficient | Pr(>\|t\|) | Coefficient | Pr(>\|t\|) |
| (Intercept) | 18.863 | 3.47e-6 | 0.834 | 8.310e-12 |
| PCOA axis 1 | -5.436 | 0.666 | 0.138 | 0.581 |
| PCOA axis 2 | 7.509 | 0.610 | 0.539 | 0.092 |

| Jaccard | Response: SMI | | Response: n° fledglings/n° nestlings | |
| --- | --- | --- | --- | --- |
| Explan. var. | Coefficient | Pr(>\|t\|) | Coefficient | Pr(>\|t\|) |
| (Intercept) | 18.863 | 3.54e-6 | 0.834 | 9.490e-12 |
| PCOA axis 1 | -2.312 | 0.847 | 0.111 | 0.643 |
| PCOA axis 2 | -8.103 | 0.551 | -0,495 | 0.104 |

| Weighted UniFrac | Response: SMI | | Response: n° fledglings/n° nestlings | |
| --- | --- | --- | --- | --- |
| Explan. var. | Coefficient | Pr(>\|t\|) | Coefficient | Pr(>\|t\|) |
| (Intercept) | 18.863 | 3.54e-6 | 0.834 | 2,41e-11 |
| PCOA axis 1 | -2.312 | 0.847 | 0.624 | 0.452 |
| PCOA axis 2 | -8.103 | 0.551 | -0.523 | 0.575 |

| Unweighted Unifrac | Response: SMI | | Response: n° fledglings/n° nestlings | |
| --- | --- | --- | --- | --- |
| Explan. var. | Coefficient | Pr(>\|t\|) | Coefficient | Pr(>\|t\|) |
| (Intercept) | 18.863 | 2.29e-6 | 0.834 | 1.530e-11 |
| PCOA axis 1 | -19.172 | 0.405 | -0.457 | 0.296 |
| PCOA axis 2 | -25.068 | 0.370 | 0.697 | 0.378 |
